# Circulating Non-Coding RNAs as Liquid Biopsy Biomarkers for Early Detection of Triple-Negative Breast Cancer: From Diagnostic Performance to Clinical Translation

**DOI:** 10.3390/diagnostics16213591

**Published:** 2026-10-30

**Authors:** Ivayla Kuleva, Stoyan Stoyanov, Maria Tsvetkova, Nikoleta Ivanova, Dzheni Cherneva, Tsonka Dimitrova, Galina Yaneva

**Affiliations:** Department of Biology, Faculty of Pharmacy, Medical University of Varna, 84 Tzar Osvoboditel Blvd., 9002 Varna, Bulgaria; stoyan.stoyanov@mu-varna.bg (S.S.); maria.cvetkova@mu-varna.bg (M.T.); nikoleta.ivanova@mu-varna.bg (N.I.); djeni.cherneva@mu-varna.bg (D.C.); tsonka.dimitrova@mu-varna.bg (T.D.); galina.yaneva@mu-varna.bg (G.Y.)

**Keywords:** ncRNA, non-coding RNA, miRNA, microRNA, lncRNA, long non-coding RNA, circRNA, circular RNA, TNBC, triple-negative breast cancer

## Abstract

**Background/Objectives**: Triple-negative breast cancer (TNBC) is an aggressive and molecularly heterogeneous breast cancer subtype for which early detection remains clinically important. Circulating non-coding RNAs (ncRNAs), including microRNAs (miRNAs), long non-coding RNAs (lncRNAs), and circular RNAs (circRNAs), are being investigated as blood-based biomarkers. This narrative review evaluates the clinical evidence for circulating ncRNAs as liquid-biopsy biomarkers for early TNBC detection, focusing on stage I–II disease, diagnostic performance, comparator selection, and validation. **Methods**: Peer-reviewed studies published between 2009 and October 2026 were identified primarily through PubMed/MEDLINE searches, supplemented by Google Scholar searches, with particular attention to disease stage, comparator populations, diagnostic performance, and validation status. **Results**: Among the ncRNA classes reviewed, circulating miRNAs provide the most directly relevant, although still limited, clinical evidence for early-stage TNBC detection, whereas evidence for lncRNAs and circRNAs remains predominantly exploratory and insufficiently validated for early-stage detection. Translation is limited by small, heterogeneous cohorts, analytical variability, and insufficient independent and prospective validation. **Conclusions**: High diagnostic accuracy in selected cohorts does not establish clinical readiness. Standardized analytical workflows, independent multicenter validation, prospective evaluation in intended-use populations, and integration with imaging and clinical assessment are needed before circulating ncRNA signatures can be considered for clinical use.

## 1. Introduction

Breast cancer remains a major global public health challenge and is the most frequently diagnosed malignancy among women worldwide. According to GLOBOCAN 2024 estimates from the International Agency for Research on Cancer, approximately 2.43 million new female breast cancer cases and 694,000 female breast cancer deaths occurred globally in 2024, with breast cancer accounting for 24.3% of all newly diagnosed cancers among women [1,2]. Furthermore, approximately 8.16 million people worldwide were living with a breast cancer diagnosis made within the preceding five years, reflecting the substantial global burden of the disease [2].

Triple-negative breast cancer (TNBC) accounts for approximately 10–15% of breast cancers and is clinically defined by the absence of estrogen receptor and progesterone receptor expression and human epidermal growth factor receptor 2 (HER2) overexpression or amplification [3]. TNBC remains a major clinical challenge because of its aggressive clinical behavior and historically limited availability of subtype-specific therapeutic targets [4]. Importantly, TNBC does not represent a single biological entity but encompasses tumors with substantial histological, genomic, and immunological heterogeneity [5,6]. This heterogeneity reflects the diverse molecular and pathological mechanisms underlying TNBC and has important implications for disease classification and therapeutic response [7]. Although the treatment landscape has expanded with the introduction of immune checkpoint inhibitors, poly(ADP-ribose) polymerase inhibitors, and antibody–drug conjugates [8], the prognosis of patients with metastatic TNBC remains substantially worse than that of patients diagnosed at an earlier stage [3]. The tendency of TNBC toward early metastatic relapse and the poor prognosis associated with advanced disease further emphasize the clinical importance of timely detection [9]. These characteristics continue to drive the search for biomarkers capable of improving early detection and molecular characterization of the disease.

Early detection of TNBC remains challenging due to its aggressive biological behavior, rapid tumor progression, and distinct imaging characteristics. TNBC is overrepresented among interval cancers diagnosed between scheduled mammographic screening examinations: it accounted for 21% of interval-detected but only 6% of screening-detected cancers in the study by Ambinder et al. [10], and in a high-risk screening cohort all three interval cancers were triple-negative [11]. This suggests that its rapid growth may shorten the preclinical window during which tumors can be detected through routine screening. Furthermore, TNBC lesions may exhibit mammographic features that are less typical of malignancy, frequently presenting as masses without suspicious microcalcifications and sometimes displaying round or oval shapes and circumscribed margins that may resemble benign lesions, potentially complicating their early recognition [12,13,14]. These biological and imaging-related challenges highlight the need to investigate complementary diagnostic approaches, including circulating non-coding RNAs as potential liquid-biopsy biomarkers, although their clinical utility for early TNBC detection has not yet been established.

Liquid biopsy has emerged as a minimally invasive strategy for assessing tumor-associated material released into blood and other body fluids. Its principal analytes include circulating tumor DNA, circulating tumor cells, extracellular vesicles (EVs), and circulating RNA species. The possibility of repeated sampling makes liquid biopsy particularly attractive for biologically heterogeneous tumors, because it allows longitudinal assessment without the need for repeated tissue biopsy [15]. In TNBC, liquid-biopsy approaches are being investigated for diagnosis, treatment monitoring, detection of residual disease, and identification of recurrence [16,17]. This expanding role is also reflected in recent TNBC-focused liquid-biopsy reviews [8,18,19].

Among the different liquid-biopsy analytes, circulating non-coding RNAs (ncRNAs) have attracted considerable interest as potential biomarkers [16,20]. The best studied are microRNAs (miRNAs), while long non-coding RNAs (lncRNAs) and circular RNAs (circRNAs) are increasingly being investigated. Circulating miRNAs can be detected in serum, plasma, whole blood, extracellular vesicles, and other biofluids [8], whereas the analytical standardization of circulating lncRNAs [21] and circRNAs [22] remains limited. Their biological relevance is supported by their involvement in gene regulation, epithelial–mesenchymal transition, metastatic progression, stemness, and treatment resistance [23,24].

Circulating ncRNAs comprise three main categories discussed in this review. MicroRNAs are short, approximately 22-nucleotide RNAs that regulate gene expression post-transcriptionally and can act as oncogenic or tumor-suppressive regulators. In TNBC, altered circulating levels of miR-21, miR-155, and miR-205 have been reported [25], while miR-105 and miR-93-3p have been implicated in chemoresistance [26]. Long non-coding RNAs are transcripts longer than 200 nucleotides that regulate chromatin state, transcription, and signaling. In breast cancer, HOTAIR has been implicated in metastasis [27], whereas specific GAS5 transcripts have been shown to promote apoptosis in breast epithelial and breast cancer cell lines, while GAS5 expression is reduced in breast ductal carcinoma tissue compared with adjacent non-malignant tissue [28]. In TNBC, lncRNA T376626 has been reported to promote proliferation, migration, and invasion [29]. Circular RNAs are covalently closed RNA molecules that can act as miRNA sponges [30]. In TNBC, exosomal circPSMA1 has been implicated in tumor progression [31]. These biological findings do not, by themselves, establish diagnostic utility.

The available evidence for TNBC-specific circulating ncRNA biomarkers is unevenly distributed across ncRNA classes [16]. Throughout this review, diagnostic discrimination refers to the ability of a circulating biomarker to distinguish patients with TNBC from a specified comparator group (healthy women, benign breast disease, or other breast cancer subtypes), usually quantified by the area under the receiver operating characteristic curve (AUC); the comparator group therefore defines the diagnostic scenario. Circulating miRNAs have been investigated most extensively and currently provide the most direct evidence for diagnostic discrimination in early-stage TNBC, including studies specifically evaluating stage I–II disease [25,26,32]. Circulating lncRNAs have also shown promising diagnostic performance, although most studies have focused on overall TNBC diagnosis or discrimination from other breast cancer subtypes rather than dedicated early-stage detection [33,34]. Evidence for circulating circRNAs remains more preliminary and is frequently derived from small clinical cohorts combined with mechanistic investigations [31,35,36]. Collectively, these differences suggest that the strength of the available evidence for early TNBC detection varies substantially according to ncRNA class and, importantly, according to whether the intended endpoint is general TNBC diagnosis or specifically early-stage disease detection.

However, high diagnostic performance in case–control studies should not automatically be interpreted as evidence of clinically useful early detection. Comparisons between patients with established TNBC and healthy individuals represent a substantially less demanding diagnostic task than distinguishing early-stage TNBC from benign breast disease or from other breast cancer molecular subtypes [37,38]. The latter comparisons are more relevant to real-world diagnostic practice, yet they remain uncommon in the TNBC liquid-biopsy literature [39]. In addition, available studies differ substantially in specimen type, analytical platform, normalization strategy, cohort size, stage composition, and validation design. Independent external validation is also limited for many proposed signatures [39].

Existing reviews have addressed related but distinct questions. Broad overviews cover liquid biopsy in breast cancer across several analytes, including circulating tumor cells, circulating tumor DNA, cell-free RNA, and exosomes [15], whereas the TNBC-specific overview by Mazzeo et al. focuses mainly on prognostic and predictive applications, such as relapse detection and treatment monitoring [16]. A review of blood circulating ncRNAs in TNBC surveyed miRNAs, lncRNAs, and circRNAs for diagnosis, prediction, and prognosis, but it was not specifically focused on stage I–II detection, and circulating circRNA evidence in TNBC was still very limited at that time [20]. A recent scoping review mapped protein-, RNA-, and DNA-based liquid-biopsy biomarkers for the diagnosis of TNBC up to 2024 [39]. Other reviews concentrate on circulating miRNAs in breast cancer across subtypes [8], on miRNA and lipidomic biomarkers for early breast cancer detection [40], or on circRNAs in breast cancer as a whole, including their biogenesis, tissue and circulating biomarker roles, and treatment resistance, rather than on early detection of TNBC [22,41]. The present review is therefore distinguished by its analytical focus rather than by its scope alone: it examines circulating miRNAs, lncRNAs, and circRNAs together, with emphasis on the evidence for early detection of TNBC, the diagnostic accuracy reported, the choice of comparator populations and validation strategy, and the limitations that currently constrain clinical application.

Therefore, this review critically evaluates the available clinical evidence for circulating miRNAs, lncRNAs, and circRNAs as liquid-biopsy biomarkers for early TNBC detection, with particular emphasis on stage I–II representation, comparator selection, diagnostic performance, validation strategies, and the methodological barriers that currently limit their translation into clinical practice.

**Figure 1:**
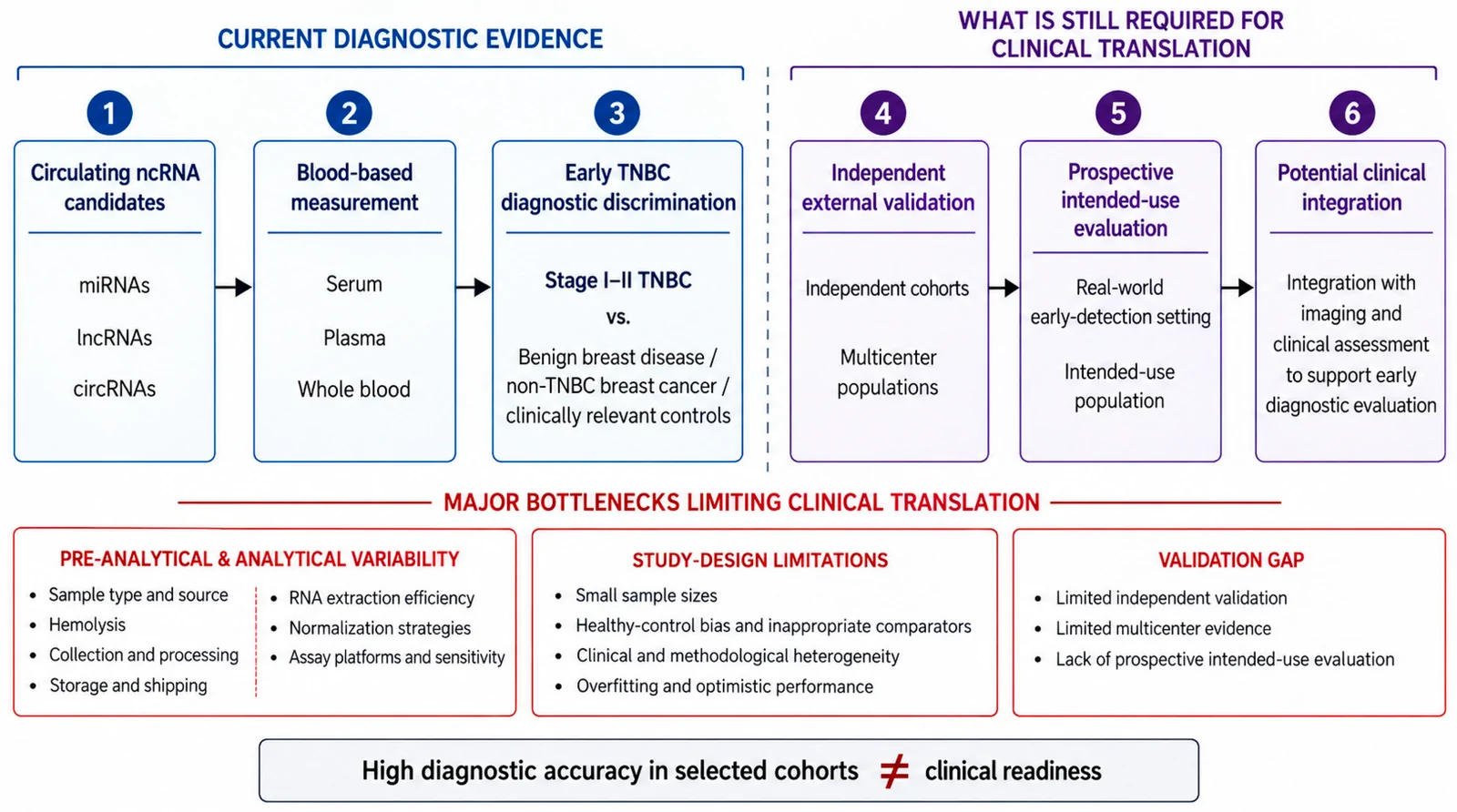
Translational pathway and major bottlenecks for circulating ncRNA biomarkers in early TNBC detection. Current evidence supports blood-based detection and diagnostic discrimination, whereas clinical translation requires independent external validation, prospective evaluation in intended-use populations, and integration with clinical assessment. Major barriers include methodological variability, study-design limitations, and insufficient validation. This figure was generated using ChatGPT (Version 6, OpenAI).

The conceptual framework used to evaluate the evidence and translational readiness of circulating ncRNA biomarkers for early TNBC detection is summarized in Figure 1.

## 2. Methods

### 2.1. Search Strategy

A narrative literature review was conducted to evaluate the available evidence on circulating non-coding RNAs (ncRNAs) as potential biomarkers for the early detection of triple-negative breast cancer (TNBC) using liquid biopsy.

The literature search covered peer-reviewed publications published between 2009 and October 2026. PubMed/MEDLINE served as the primary database, supplemented by Google Scholar.

The principal search strategy combined terms related to TNBC, non-coding RNAs, liquid biopsy, and diagnostic applications: (“triple-negative breast cancer” OR “TNBC”) AND (“non-coding RNA” OR “noncoding RNA” OR “miRNA” OR “microRNA” OR “lncRNA” OR “long non-coding RNA” OR “circRNA” OR “circular RNA”) AND (“circulating” OR “plasma” OR “serum” OR “blood” OR “liquid biopsy”) AND (“diagnosis” OR “diagnostic” OR “early detection” OR “biomarker”). Additional targeted searches were performed for individual ncRNA classes to identify potentially relevant studies not retrieved by the principal search strategy. Where applicable, searches were restricted to English-language publications. Original research articles, review articles, and meta-analyses were considered during the initial search to identify relevant primary studies and synthesize the existing evidence. The search strategy was adapted to the search functions and indexing characteristics of each source.

### 2.2. Scope and Selection of Evidence

The literature search was primarily directed toward original clinical studies investigating circulating ncRNAs (in blood, plasma, serum, or extracellular vesicles) as potential diagnostic biomarkers in patients with TNBC. Particular attention was given to studies reporting diagnostic performance measures, including the area under the receiver operating characteristic curve (AUC), sensitivity, and specificity, especially those involving patients with stage I–II or explicitly defined early-stage TNBC.

Studies conducted in broader breast cancer populations were used as supplementary evidence when they provided relevant information on circulating ncRNA detection, diagnostic performance, or biomarker validation. Their findings were not interpreted as direct evidence of TNBC-specific early detection. Reviews and meta-analyses, including those addressing breast cancer in general, were not considered primary evidence; they were used to provide background and context and to identify additional primary studies.

Studies without relevant measurements of circulating biomarkers were not considered primary diagnostic evidence. Similarly, studies addressing only prognosis, treatment response, or recurrence monitoring without a relevant diagnostic component were not the focus of the diagnostic synthesis. Such studies were considered only when they provided relevant biological or clinical context. Exploratory studies combining circulating ncRNA measurements in patient samples with mechanistic investigations were considered separately and distinguished from studies establishing diagnostic performance.

### 2.3. Graphical Presentation of Data

Microsoft Excel 2016 was used for the graphical presentation of the collected data. The figures included in this manuscript were generated using ChatGPT (Version 6, OpenAI).

## 3. Results

The initial literature search identified 59 publications, of which 40 were retained for the narrative synthesis following an assessment of their relevance to the review objectives. The remaining 19 publications were not retained because they did not provide relevant diagnostic evidence on circulating ncRNAs or meaningful supporting biological or methodological context. These included publications addressing other malignancies without relevant diagnostic or biological implications for TNBC, as well as studies focusing on prognosis, treatment response, or disease recurrence that provided neither relevant diagnostic evidence nor meaningful supporting context for the review objectives. The publications considered for the narrative synthesis included original studies of circulating ncRNAs in human samples, reviews and meta-analyses, and tissue-based or mechanistic studies used for background and context. Of these, 11 original diagnostic and exploratory studies investigating circulating ncRNAs in relation to TNBC were selected for comparative presentation in Table 1. The selected publications were qualitatively synthesized according to ncRNA type, biological specimen, study population, comparator group, disease stage, analytical method, reported diagnostic performance, and validation strategy.

### 3.1. Circulating miRNAs as Diagnostic Biomarkers for TNBC

Among circulating non-coding RNAs, miRNAs have been the most extensively evaluated as blood-based diagnostic biomarkers for TNBC [16]. Several clinical studies have demonstrated discriminatory performance not only between TNBC and healthy controls, but also between TNBC and other breast cancer subtypes, with some studies specifically addressing early-stage disease [25,26,32,42].

Meta-analyses evaluating circulating miRNAs and other blood-based biomarkers in breast cancer have reported promising diagnostic performance, providing a broader rationale for investigating their potential in early-stage TNBC [40,43]. However, these pooled estimates were derived from breast cancer in general and were not specific to TNBC. Their interpretation is limited by substantial between-study heterogeneity, the retrospective or prospective case–control designs of the included studies, differences in comparator populations and case-to-control ratios, variability in normalization procedures, and, in one meta-analysis, the use of a validation cohort in only about 40% of the included studies [43]. Consequently, these findings cannot be directly extrapolated to early TNBC detection.

Among the studies that specifically evaluated stage I–II or explicitly defined early-stage TNBC, circulating miRNA signatures reached AUC values of 0.814–0.949, with sensitivity of 83.8–91.0% and specificity of 74.2–82.3% where these were reported [25,26,32]. The two early-stage analyses with a stated comparator (Kumar et al. and Kahraman et al.) used healthy controls as the reference group, whereas the comparator for the stage I/II subgroup reported by Li et al. was not specified. None of these early-stage estimates were reported against women with benign breast disease. Diagnostic accuracy therefore appears consistently good, but it varied with signature size, specimen type, and comparator population, as detailed below.

Shin et al. evaluated circulating miRNAs in plasma from patients with TNBC, non-TNBC breast cancer, and healthy controls. Following an initial miRNA profiling phase and subsequent validation, miR-16, miR-21, and miR-199a-5p were found to be significantly underexpressed in TNBC compared with non-TNBC breast cancer [42]. Among these candidates, plasma miR-199a-5p showed the strongest diagnostic performance, with an AUC of 0.8838 for discriminating TNBC from healthy controls and non-TNBC patients. Its expression was also associated with tumor–node–metastasis (TNM) stage and tumor subtype, while postoperative levels of miR-16, miR-21, and miR-199a-5p were significantly restored compared with preoperative levels [42].

Evidence specifically relevant to early-stage disease was provided by Li et al. [26], who evaluated circulating miR-105 and miR-93-3p in plasma samples from 118 breast cancer patients, including 74 TNBC and 44 non-TNBC cases, together with 12 healthy controls. Both miRNAs were elevated in TNBC compared with non-TNBC breast cancer. Compared with healthy controls, the difference was significant for miR-105 (*p* = 0.014) but not for miR-93-3p (*p* = 0.057). In receiver operating characteristic (ROC) analysis, circulating miR-105 and miR-93-3p distinguished TNBC from non-TNBC breast cancer, with AUCs of 0.928 (95% confidence interval [CI] 0.880–0.975) and 0.657 (95% CI 0.554–0.759), respectively. In a stage I/II subgroup (*n* = 46), the combined miR-105/93-3p signature reportedly achieved an AUC of 0.939 (95% CI 0.868–1.000), compared with 0.986 (95% CI 0.947–1.000) in stage III/IV disease (*n* = 21) [26]. The comparator group for these stage-stratified analyses was not stated by the authors.

Kumar et al. [25] subsequently evaluated serum miR-205, miR-155, and miR-21 in 139 patients with TNBC and 51 healthy controls. miR-155 and miR-21 were significantly upregulated, whereas miR-205 was downregulated in TNBC. Importantly, the study separately assessed 78 patients with stage I–II TNBC. The combined three-miRNA signature achieved an AUC of 0.949, with 91.0% sensitivity and 82.3% specificity, for discriminating stage I–II TNBC patients from healthy controls; the corresponding AUC values were 0.961 in the overall TNBC cohort and 0.971 in stage III–IV disease [25]. These findings provide direct evidence that circulating miRNA combinations can retain high discriminatory capacity in stage I–II TNBC, although the use of healthy individuals as the comparator group limits conclusions regarding performance in a clinically representative diagnostic setting.

Kahraman et al. [32] also specifically investigated blood-borne miRNA signatures in early-stage basal-like TNBC. Genome-wide profiling was initially performed in 21 patients and 21 age-matched healthy controls, followed by validation by reverse transcription quantitative polymerase chain reaction (RT-qPCR). A seven-miRNA signature comprising miR-126-5p, miR-144-5p, miR-144-3p, miR-301a-3p, miR-126-3p, miR-101-3p, and miR-664b-5p achieved an average cross-validated AUC of 0.814, with 83.8% sensitivity, 74.2% specificity, and 79% accuracy for discriminating early-stage basal-like TNBC patients from healthy controls. The study additionally demonstrated changes in circulating miRNA profiles following neoadjuvant chemotherapy, indicating potential complementary applications in treatment monitoring [32].

Extracellular-vesicle-associated miRNAs provide an additional source of circulating biomarkers. Ozawa et al. [44] identified serum EV-associated miRNAs by RNA sequencing followed by RT-qPCR validation. A panel comprising miR-142-5p, miR-320a, and miR-4433b-5p distinguished breast cancer patients from healthy controls with an AUC of 0.8387, although the reported diagnostic model was not specific to TNBC. Thus, despite inclusion of TNBC samples, these results provide supportive rather than direct evidence for TNBC-specific early detection [44].

An earlier paired tissue–serum study examined six miRNAs known to be involved in breast carcinogenesis in 85 breast cancer patients, 23 of whom had TNBC, together with healthy women and patients with benign fibroadenoma [45]. In TNBC, the oncogenic miR-21, miR-221, and miR-210 were upregulated in both tumor tissue and serum, whereas the tumor suppressors miR-195 and miR-145 were downregulated; the tumor suppressor let-7a was unexpectedly upregulated. Serum changes mirrored those in tissue, although at a lower magnitude, and, within the TNBC subgroup, were associated with tumor grade, clinical stage, and proliferative activity (Ki-67). These findings associate the circulating miRNA profile with altered expression of miRNAs that have previously been implicated in oncogenic or tumor-suppressive processes.

Qattan et al. [46] measured miRNAs in pretreatment plasma from 36 patients with TNBC, 57 patients with luminal tumors (cohorts described by the authors as early-stage diagnoses), and 34 disease-free women. Eighteen miRNAs were differentially present; let-7 family miRNAs were elevated in patients of both subtypes, and the tumor suppressor miR-195 was elevated in the TNBC group. Comparison with data from The Cancer Genome Atlas showed that miR-195 and let-7 were poorly expressed in tumor tissue but strongly expressed in adjacent non-tumor tissue, suggesting that cancer cells may selectively export these tumor-suppressor miRNAs. The increase in plasma miR-195 contrasts with the decreased serum miR-195 reported in the paired tissue–serum study above [45], illustrating the inconsistency of circulating miRNA directions across cohorts and platforms.

Supporting evidence comes from broader early-stage breast cancer cohorts that were not restricted to TNBC. In a serum small-RNA sequencing study by Koi et al., a model based on three small RNAs (an isomiR of miR-21-5p, miR-23a-3p, and a tRNA-derived fragment) distinguished 78 patients with stage 0–III breast cancer from 72 cancer-free controls with an AUC of 0.92 and also discriminated stage 0 disease from controls [47]. An eight-miRNA serum panel developed by Zou et al. in a multicenter case–control design reached an AUC of 0.915 in a locked validation set and 0.916 in stage I–II breast cancer, again against non-cancer controls [48]. In an extracellular-vesicle study by Kim et al., a four-miRNA panel (miR-9, miR-16, miR-21, and miR-429) measured in tumor-derived vesicles from the plasma of 62 breast cancer patients (55 with early-stage disease) discriminated each subtype from 20 healthy controls, with AUCs of 0.90 (luminal A), 0.86 (luminal B), 0.88 (HER2-positive), and 0.84 (triple-negative, *n* = 15) [49]. However, these studies compared patients with healthy or cancer-free individuals and were not designed as dedicated validation studies for stage I–II TNBC detection.

### 3.2. Circulating lncRNAs as Diagnostic Biomarkers for TNBC

Circulating lncRNAs have also demonstrated diagnostic potential in TNBC, although the clinical evidence is less extensive and considerably less developed for stage I–II disease [29,33,34].

Liu et al. [33] performed plasma lncRNA profiling in patients with TNBC and non-TNBC breast cancer and identified ANRIL, HIF1A-AS2, and UCA1 as significantly upregulated in TNBC. Individually, these lncRNAs demonstrated moderate diagnostic discrimination, with AUCs of 0.785 for ANRIL, 0.739 for HIF1A-AS2, and 0.817 for UCA1 when differentiating TNBC from non-TNBC breast cancer. A combined three-lncRNA score, TNBCSigLnc-3, substantially improved diagnostic performance, reaching an AUC of 0.934, with 76.0% sensitivity and 97.1% specificity [33]. However, stage-specific diagnostic performance was not reported.

More recently, He et al. [29] identified circulating lncRNA T376626 through genome-wide serum microarray screening. In a validation cohort of 282 breast cancer patients (72 with TNBC) and 78 healthy controls, with an additional group of 70 patients with benign breast disease, serum T376626 levels were significantly higher in TNBC than in non-TNBC breast cancer, benign breast disease, and healthy controls. T376626 distinguished TNBC from healthy controls with an AUC of 0.842 (sensitivity 73.7%, specificity 87.5%). Across the overall breast cancer cohort, serum expression was associated with histological type (particularly medullary carcinoma), Ki-67 status, and molecular subtype, but not with age, tumor size, or TNM stage. In a separate analysis of 48 TNBC serum samples, T376626 did not distinguish early- from late-stage disease (AUC 0.500). In breast cancer tissue, expression increased with pathological differentiation grade and was higher in HER2-positive and triple-negative tumors. Higher tissue expression was associated with shorter overall survival among 114 breast cancer patients and among 29 patients with TNBC; its independent prognostic association was evaluated in the overall 114-patient cohort. A dedicated diagnostic analysis of stage I–II TNBC versus healthy controls was not reported, and the study was conducted at a single center [29].

Guler et al. [50] evaluated HOTAIR, HULC, ANRIL, NEAT1, MALAT1, and AFAP1-AS1 in primary tumor tissue and serum exosomes of 21 patients with sporadic TNBC and in 30 healthy controls. HOTAIR, HULC, ANRIL, and AFAP1-AS1 were downregulated, whereas NEAT1 and MALAT1 were upregulated, in patients compared with controls; for HOTAIR, HULC, and ANRIL, the direction of change was consistent between tissue and serum. Notably, HOTAIR was decreased in this cohort, in contrast to its increased expression in primary breast tumors reported earlier [27]. All six lncRNAs discriminated TNBC patients from healthy controls, with AUC values of 1.000 for HOTAIR and ANRIL and between 0.960 and 0.990 for the remaining candidates [50]. However, these exceptionally high diagnostic estimates were obtained from a small cohort without an independent validation population, and stage I–II-specific diagnostic performance was not established.

Serum TINCR was measured before treatment in 72 patients with TNBC, 105 with non-TNBC breast cancer, 60 with benign breast disease, and 86 healthy subjects [34]. Levels were higher in breast cancer than in benign disease or healthy controls and highest in TNBC, and TINCR distinguished TNBC from non-TNBC breast cancer with an AUC of 0.797. The study was primarily prognostic. In TNBC, higher serum TINCR was associated with larger tumor size, lymph node metastasis, advanced TNM stage, and higher histological grade. Patients with higher TINCR expression had poorer overall and recurrence-free survival, and TINCR remained an independent prognostic factor for overall survival in multivariate analysis (hazard ratio 2.540, 95% CI 1.348–5.527), whereas no significant association with survival was found in non-TNBC. These prognostic and clinicopathological associations do not establish early-detection utility; the diagnostic comparison was limited to TNBC versus non-TNBC and was not stage-specific.

Serum exosomal XIST discriminated 91 patients with stage II–III TNBC from 50 healthy controls (AUC 0.888, 95% CI 0.814–0.935; sensitivity 84.2%, specificity 92.3%) [51]. Beyond this diagnostic comparison, XIST levels were significantly higher in the 25 patients who developed recurrence than in the 66 who did not, decreased after resection of the primary tumor, and increased again at recurrence, with no association with age or stage. High serum exosomal XIST was also associated with poorer overall survival (hazard ratio 3.54, 95% CI 1.55–5.45). Although the cohort included stage II patients, it did not include stage I disease or provide a separate diagnostic analysis restricted to stage I–II TNBC. The longitudinal findings primarily support the potential relevance of XIST for recurrence monitoring and prognosis.

LncRNA dysregulation is not restricted to TNBC. Transcripts such as ANRIL are also upregulated in other malignancies, including lung cancer and hepatocellular carcinoma [52,53]. In TNBC, LINC01559 and MIR100HG have been reported to be upregulated in tumor tissues compared with adjacent non-tumor tissues, based on analyses of 96 and 82 paired samples, respectively [54,55]. Importantly, lncRNAs may also exert tumor-suppressive functions. In the cohort studied by Zhang et al. (2023), PTCSC3 was downregulated, and its experimental overexpression reduced cell viability and promoted apoptosis; similarly, LINC00993 has been shown to suppress TNBC growth in vitro and in vivo [55,56]. These findings highlight the context-dependent roles of lncRNAs and the need to distinguish tissue-based evidence from the diagnostic performance of circulating biomarkers.

Circulating lncRNA expression does not necessarily mirror expression in the corresponding tumor tissue, and the relationship between these compartments appears to be transcript-dependent. In patients with TNBC, Liu et al. [33] reported a significant positive correlation between circulating and tissue HIF1A-AS2 expression (*r* = 0.6702, *p* = 0.0002), whereas the corresponding correlations for ANRIL (*r* = 0.3954, *p* = 0.0504) and UCA1 (*r* = 0.3522, *p* = 0.0842) did not reach statistical significance. In breast cancer more broadly, Zhang et al. (2018) demonstrated a moderate positive correlation between plasma and tumor tissue lincRNA-ROR expression in 24 matched samples (*r^2^* = 0.638, *p* < 0.0001) [57]. More recently, Guler et al. (2026) [50] investigated tissue- and serum-derived exosomal lncRNAs in 21 patients with sporadic TNBC. Although HOTAIR, HULC, and ANRIL exhibited concordant downregulation relative to their respective controls, statistically significant differences in their measured expression levels were reported between the tissue and serum compartments, even though the authors interpreted the overall expression patterns as consistent. However, concordant directions of differential expression do not necessarily establish patient-level correlation, and differences in measured expression levels should not be interpreted as opposite directions of regulation. Collectively, these findings indicate that circulating lncRNA levels cannot automatically be considered reliable surrogates for tumor tissue expression. Larger studies using matched blood and tumor samples, standardized analytical methods, and transcript-specific correlation analyses are required to establish the clinical significance of blood–tissue expression relationships.

### 3.3. Circulating circRNAs and Exosomal circRNAs

Circular RNAs (circRNAs) are a distinct class of RNA molecules characterized by covalently closed circular structures that lack free 5′ and 3′ ends. They are predominantly generated through back-splicing, and their circular configuration confers increased resistance to exonuclease-mediated degradation compared with linear RNA transcripts [30,58,59]. CircRNAs participate in the regulation of gene expression and cancer-related cellular processes, including proliferation, apoptosis, and metastasis, while their relative stability and detectability in biological fluids have stimulated interest in their potential as circulating biomarkers [59,60,61].

In breast cancer, circRNA dysregulation has been linked to tumor progression and has supported growing interest in their potential utility as biomarkers [41]. Their covalently closed structure, stability in body fluids, and potential detection in plasma further strengthen their relevance to liquid-biopsy applications in breast cancer [22]. Clinical evidence for circulating circRNAs specifically in TNBC nevertheless remains comparatively limited.

Darbeheshti et al. [35] investigated circ_0000977 as a potential circulating biomarker in TNBC following its identification as a dysregulated circRNA in TNBC models. Downregulation was demonstrated in TNBC cell lines and tumor tissues and was subsequently evaluated in plasma samples from patients with TNBC and healthy controls. The study also identified a putative circ_0000977/miR-135b-5p/APC/GATA3 regulatory network, providing mechanistic support for its association with TNBC. However, the circulating biomarker analysis was based on a small clinical cohort, and stage I–II-specific diagnostic performance and independent external validation were not established [35].

Yang et al. [31] demonstrated increased circPSMA1 expression in TNBC cells, tumor-derived exosomes, and serum exosomes from patients with TNBC. Functional experiments indicated that exosomal circPSMA1 promotes proliferation, migration, and metastatic behavior through the miR-637/Akt1/β-catenin signaling axis. Although these findings support the detectability and biological relevance of circulating exosomal circPSMA1 in TNBC, the study was primarily mechanistic and did not establish a validated diagnostic model for stage I–II TNBC detection [31].

More recently, Ji et al. [36] reported elevated circSTIL (hsa_circ_0000069) in TNBC cell lines and plasma exosomes. Plasma exosomal circSTIL showed potential for distinguishing TNBC from non-TNBC breast cancer and was associated with adverse clinical characteristics. However, the study primarily investigated its relationship with pirarubicin response, and no independently validated stage I–II diagnostic model was established. Accordingly, its relevance to early TNBC detection remains preliminary [36].

Additional evidence comes from broader breast cancer cohorts that were not restricted to TNBC; in each of these studies, patients were compared with healthy controls. Plasma hsa_circ_0001785 distinguished breast cancer patients from healthy volunteers with an AUC of 0.784 in an extended cohort of 57 participants, outperforming carcinoembryonic antigen (AUC 0.562) and cancer antigen 15-3 (AUC 0.629), and its level was associated with histological grade, TNM stage, and distant metastasis [62]. Peripheral blood hsa_circ_0104824, which was downregulated in breast cancer, discriminated 83 patients from 49 healthy persons with an AUC of 0.849 and was associated with tumor size, TNBC status, and estrogen and progesterone receptor status [63]. Plasma circ_0042881, which was upregulated in breast cancer tissue and associated with more advanced TNM stage and larger tumor size, discriminated 74 patients with breast cancer from 46 healthy donors with an AUC of 0.802 [64]. Yu et al. [65] developed a three-circRNA plasma model (hsa_circ_0000091, hsa_circ_0067772, and hsa_circ_0000512) in 202 patients with breast cancer, most of whom had stage I–II disease (*n* = 165), and 202 healthy controls. The model achieved an AUC of 0.974 in both the training set and an internal test set. Although the cohort included 25 patients with TNBC, diagnostic performance was not evaluated separately for TNBC or stage I–II breast cancer. None of these studies was designed to establish diagnostic performance specifically in stage I–II TNBC.

Although circRNAs are relatively resistant to degradation, their circulating expression levels may change over time. In a breast cancer cohort, Mirzae et al. (2025) reported a significant decrease in circulating circRNA-000284 following chemotherapy, suggesting that treatment-related changes may influence biomarker measurements [66]. However, longitudinal evidence remains limited, particularly in TNBC, and temporal changes must be distinguished from pre-analytical variability. Standardized sample collection and repeated measurements in longitudinal cohorts are therefore needed to establish the reproducibility and clinical interpretability of circulating circRNA biomarkers.

### 3.4. Overview of Clinical Studies

**Figure 2:**
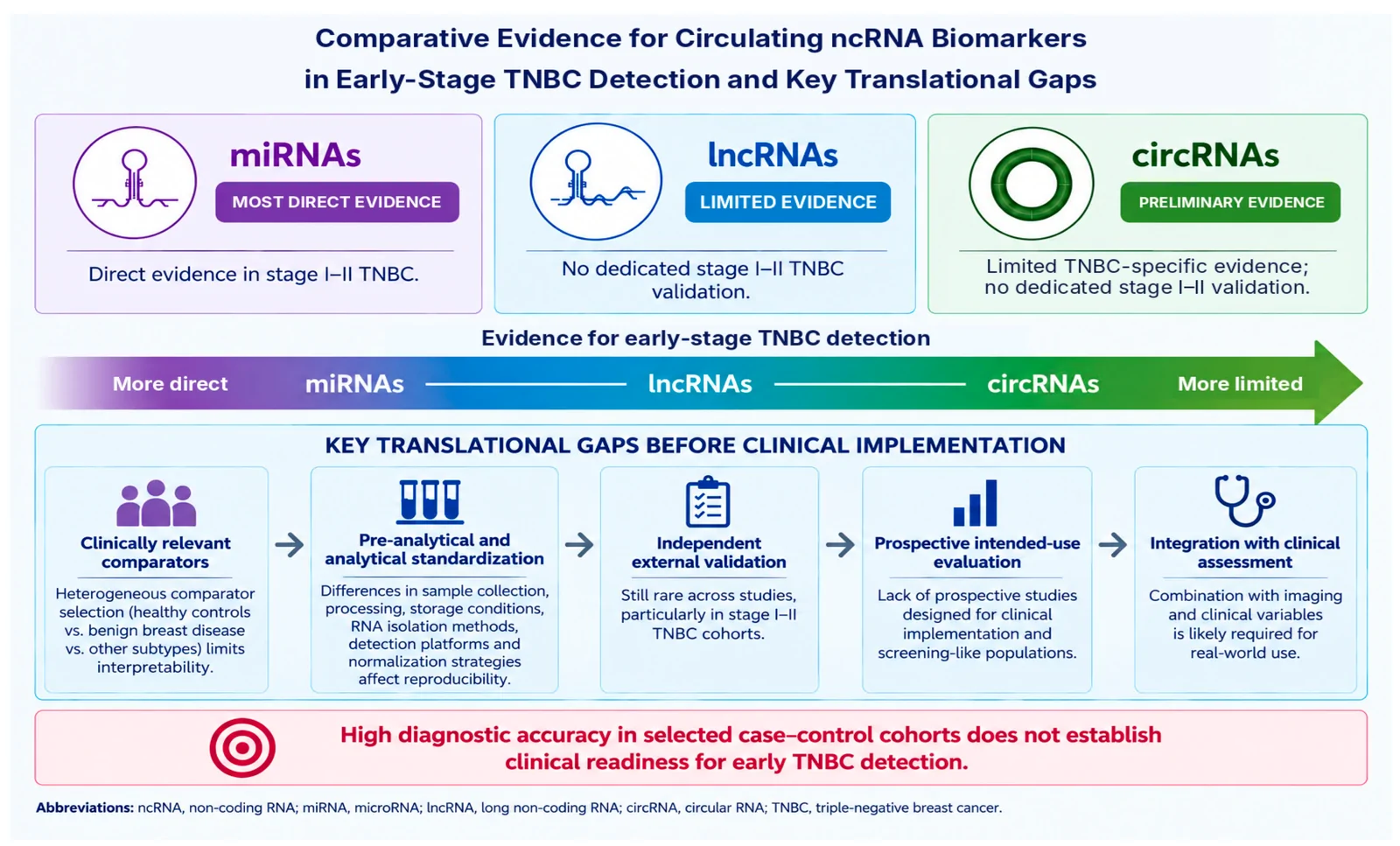
Comparative evidence for circulating ncRNA biomarkers in early-stage TNBC detection and key translational gaps. Current evidence is most directly applicable to early-stage TNBC detection for miRNAs, whereas the available evidence for lncRNAs and circRNAs is more limited. The comparison reflects the availability of studies specifically addressing early-stage TNBC detection and their validation status, rather than a formal assessment of study quality or diagnostic accuracy. Key requirements for clinical translation include clinically relevant comparators, analytical standardization, independent external validation, prospective intended-use evaluation, and integration with clinical assessment. This figure was generated using ChatGPT (Version 6, OpenAI).

The comparative evidence for circulating miRNAs, lncRNAs, and circRNAs in early-stage TNBC detection, together with the key translational gaps, is summarized in Figure 2.

The main clinical studies evaluating circulating miRNAs, lncRNAs, and circRNAs as diagnostic biomarkers in TNBC are summarized in Table 1.

**Table 1:** Clinical studies of circulating non-coding RNAs in triple-negative breast cancer, including diagnostic and exploratory studies.

Study	ncRNA Biomarker (s)	Specimen	Detection Method	Cohort/Comparator	Diagnostic Settings *	Diagnostic Performance	Validation
miRNAs							
Shin et al. [42]	miR-16, miR-21, miR-199a-5p	Plasma	miRCURY LNA array; RT-qPCR	Discovery: 5 TNBC, 5 non-TNBC, 5 HC; validation cohort *n* = 252	TNBC vs. HC; not early-stage specific	miR-199a-5p: AUC 0.884 (95% CI: 0.83–0.94)	Multi-stage validation within the study; no independent external clinical cohort.
Li et al. [26]	miR-105 + miR-93-3p	Plasma	RT-qPCR	74 TNBC, 44 non-TNBC, 12 HC	Stage I–II TNBC (*n* = 46); comparator not specified	AUC 0.939	No external validation
Kahraman et al. [32]	Seven-miRNA signature	Whole blood	Genome-wide miRNA microarray; RT-qPCR	Discovery: 21 TNBC, 21 HC; validation: 31 TNBC, 34 HC	Early-stage basal-like TNBC vs. HC	AUC 0.814 (cross-validated, discovery cohort); sensitivity 83.8%; specificity 74.2%	RT-qPCR validation (partly overlapping cohort; two laboratories); no external clinical validation
Ozawa et al. [44]	EV-miR-142-5p, miR-320a, miR-4433b-5p	Serum EVs	RNA-seq; RT-qPCR	HC, luminal A, and TNBC samples	Breast cancer vs. HC; not TNBC-specific	AUC 0.839; sensitivity 93.6%; specificity 68.8%	Screening and RT-qPCR test phases; no external validation
Kumar et al. [25]	miR-205 + miR-155 + miR-21	Serum	RT-qPCR	139 TNBC (78 stage I–II), 51 HC	Stage I–II TNBC vs. HC	AUC 0.949 (stage I–II); sensitivity 91.0%; specificity 82.3%	Internal cross-validation; no external cohort
lncRNAs							
Liu et al. [33]	ANRIL + HIF1A-AS2 + UCA1 (TNBCSigLnc-3)	Plasma	lncRNA microarray; RT-qPCR	25 TNBC, 35 non-TNBC, HC	TNBC vs. non-TNBC; not stage-specific	AUC 0.934 (95% CI 0.839–0.982); sensitivity 76.0%; specificity 97.1%	No external validation
He et al. [29]	T376626	Serum	lncRNA microarray; RT-qPCR	72 TNBC among 282 breast cancer patients; 78 healthy subjects	TNBC vs. HC; not stage-specific	AUC 0.842 (95% CI 0.771–0.912); sensitivity 73.7%; specificity 87.5%	RT-qPCR validation; no external clinical validation
Guler et al. [50]	Exosomal HOTAIR, HULC, ANRIL, NEAT1, MALAT1, AFAP1-AS1	Serum exosomes	RT-qPCR	21 TNBC, 30 HC	TNBC vs. HC; not stage-specific	HOTAIR, ANRIL: AUC 1.000; others: AUC 0.960–0.990	No independent validation
circRNAs							
Darbeheshti et al. [35]	circ_0000977	Plasma	RT-qPCR	20 TNBC, 20 age-matched HC	TNBC vs. HC; not stage-specific	AUC 0.960; sensitivity 85%; specificity 95%	No external validation
Yang et al. [31]	Exosomal circPSMA1	Serum exosomes	circRNA profiling; RT-qPCR	TNBC and non-TNBC patients; experimental models	Mechanistic; not early-stage-specific	Increased in TNBC vs. non-TNBC; no diagnostic ROC model reported	No independent clinical diagnostic validation
Ji et al. [36]	Exosomal circSTIL	Plasma exosomes	RT-qPCR	49 TNBC, 40 non-TNBC; 10 TNBC after NAC	TNBC vs. non-TNBC; not early-stage-specific	AUC 0.823 (*p* < 0.0001); sensitivity and specificity not reported	No independent external clinical validation

AUC—area under the receiver operating characteristic curve; EV—extracellular vesicle; HC—healthy controls; RNA-seq—RNA sequencing; RT-qPCR—reverse transcription quantitative polymerase chain reaction; TNBC—triple-negative breast cancer; ROC—receiver operating characteristic; NAC—neoadjuvant chemotherapy. * Note: The “Diagnostic settings” column distinguishes studies designed for early-stage TNBC detection from those addressing overall TNBC diagnosis, subtype discrimination, prognosis, treatment response, or predominantly mechanistic biomarker assessment. Reported diagnostic metrics are retained only where supported by the study information assembled for the review. AUC values are rounded to three decimal places; sensitivity and specificity values are rounded to one decimal place. Comparator refers to the control group against which the reported diagnostic performance was calculated (HC, healthy controls); where the original report did not state the comparator, this is indicated.

## 4. Discussion

The present review indicates that circulating ncRNAs represent a promising but still clinically immature class of biomarkers for the early detection of TNBC. Although several studies have reported high diagnostic performance, the strength and clinical relevance of the available evidence vary substantially according to ncRNA class, disease stage, comparator population, and validation strategy. The most direct evidence for stage I–II TNBC detection currently derives from circulating miRNAs, whereas lncRNA studies have predominantly addressed general TNBC diagnosis or subtype discrimination, and circulating circRNA studies remain largely exploratory. This interpretation is consistent with a recent scoping review of liquid-biopsy biomarkers in TNBC, which identified 32 diagnostic studies, including 12 RNA-based investigations, but concluded that the overall evidence remains heterogeneous and insufficiently validated, with no biomarker currently demonstrating reproducibility adequate for clinical implementation [39].

Integrating the evidence across the three classes, four recurring determinants explain most of the differences in how far each class has progressed toward early-detection use. First, stage specificity: the most direct diagnostic evidence for stage I–II or explicitly defined early-stage TNBC derives from circulating miRNA studies, whereas lncRNA and circRNA investigations have not established independently validated diagnostic performance specifically for this setting. Second, comparator choice: the reviewed studies employed heterogeneous comparator populations, including healthy controls, benign breast disease, and non-TNBC breast cancer, while some diagnostic estimates were derived from broader breast cancer cohorts rather than TNBC-specific comparisons. These differences limit direct comparisons of reported diagnostic performance and its relevance to early-stage TNBC detection.

Third, validation: reported performance derives largely from single, relatively small cohorts, with limited independent validation in every class. Fourth, biological interpretability: the circulating signal does not necessarily mirror the tumor, as illustrated by inconsistent directions of circulating miRNA changes across cohorts, tissue–blood differences for some lncRNAs, and treatment-related changes in circRNA levels. These shared limitations, rather than the intrinsic properties of any single RNA class, are the main reason why high point estimates of diagnostic accuracy have not yet translated into evidence of clinical readiness, and they define a common research agenda, as summarized in Figure 2.

Despite the growing number of reported circulating miRNA signatures, no consensus TNBC-specific miRNA profile has yet emerged for diagnostic use, and additional clinical validation remains necessary before these signatures can be incorporated into routine liquid-biopsy testing [16].

Among the evaluated ncRNA classes, circulating miRNAs currently provide the most direct evidence for early-stage TNBC detection, because multi-miRNA signatures were evaluated in stage I–II or explicitly early-stage populations in all three dedicated studies [25,26,32]. These studies cannot be compared directly: they differ in cohort definition, analytical strategy, comparator population and validation design, so the more moderate performance reported in one of them should not be read as weaker biomarker potential. Multi-miRNA signatures are therefore the candidates supported by the most direct early-stage TNBC diagnostic evidence, but their performance has not yet been confirmed in large independent prospective cohorts.

Recent TNBC-specific studies further illustrate the expanding biological relevance of extracellular-vesicle and circulating miRNAs, but also the distinction between biomarker discovery and clinically validated early detection. Candidate exosomal miRNAs have been identified in discovery-phase investigations [67], while circulating miRNA profiles have also been associated with recurrence risk in TNBC [68]. Neither type of evidence constitutes independent clinical validation of a circulating ncRNA test for stage I–II TNBC detection.

Importantly, circulating miRNA profiles may not directly mirror miRNA expression within the primary tumor. Discordance between plasma miRNA expression and breast tumor tissue data has been reported, highlighting the complexity of interpreting the biological origin of circulating miRNA signals [46].

However, high diagnostic accuracy does not necessarily imply equivalent clinical utility for early detection. The choice of comparator population is particularly important when interpreting circulating ncRNA studies, because discrimination between patients with established TNBC and healthy individuals represents a less clinically challenging setting than distinguishing early-stage TNBC from benign breast disease or other breast cancer subtypes. Methodological studies of diagnostic accuracy have shown that case–control designs using highly contrasting populations are susceptible to spectrum bias and may overestimate diagnostic performance, particularly when healthy controls are used instead of patients with clinically relevant alternative diagnoses [37,38]. This limitation is relevant to the TNBC liquid-biopsy literature, in which the most common comparisons have been TNBC versus healthy controls and TNBC versus other breast cancer subtypes, whereas benign breast lesions have been included less frequently [39]. Moreover, the available studies are generally small, and case–control designs predominate [39]. Consequently, diagnostic performance obtained in such settings should be interpreted cautiously when assessing the potential of circulating ncRNAs for population-level early detection.

A further limitation is the scarcity of independent external validation, which is essential for determining whether diagnostic performance is reproducible beyond the population in which a biomarker signature was developed. Several of the most promising ncRNA studies relied on single-cohort analyses, internal data partitioning, cross-validation, or partially overlapping discovery and validation samples rather than fully independent external populations. For example, the three-miRNA model evaluated by Kumar et al. used stratified cross-validation within the recruited cohort [25], while the validation strategy of the seven-miRNA signature included both technical replicates and newly recruited biological samples [32]. Although such approaches are valuable for assessing internal robustness, they cannot substitute for external validation and do not establish generalizability across independent populations and clinical settings. This distinction is particularly important for multivariable biomarker signatures developed in relatively small datasets, which are susceptible to overfitting and optimistic estimates of diagnostic performance [69,70]. Consistent with this concern, independent validation remains uncommon in the TNBC liquid-biopsy literature, and recent evidence indicates that none of the evaluated biomarkers currently demonstrates sufficient reproducibility for clinical implementation [39]. Accordingly, the high AUC values reported for several circulating ncRNA signatures should be interpreted as evidence of diagnostic potential rather than definitive evidence of clinical validity until replicated in adequately powered independent cohorts.

Another major barrier to clinical translation is the substantial pre-analytical and analytical heterogeneity across circulating ncRNA studies. Reported biomarker performance can be influenced by differences in specimen type, including serum, plasma, whole blood, and extracellular-vesicle fractions, as well as by blood collection, processing, centrifugation, storage, RNA extraction, normalization, and analytical platform. For circulating miRNAs in particular, differences in plasma preparation, RNA isolation, analytical platforms, and normalization strategies can substantially affect measured expression profiles and limit inter-study comparability [71]. Hemolysis and residual blood-cell contamination can further alter measured miRNA profiles [72]. Analytical and normalization procedures remain additional sources of inter-study variability [73]. Extracellular-vesicle analyses introduce an additional layer of methodological variability because blood collection procedures, such as the choice of anticoagulant, can influence both vesicle counts and the resulting miRNA profile [74]. Consequently, discordant biomarker results across studies may partly reflect methodological variability rather than true biological differences. Standardized pre-analytical workflows and harmonized analytical procedures are therefore essential for determining whether circulating ncRNA signatures can be reproduced across independent cohorts and ultimately translated into clinically applicable assays.

In contrast to miRNAs, circulating lncRNAs and circRNAs have more limited clinical evidence supporting their use for early TNBC detection. Although some lncRNA signatures have demonstrated promising discriminatory performance, the available studies generally involve small cohorts, heterogeneous comparator populations, limited stage-specific analyses, and insufficient independent validation [29,33,50]. Differences between circulating and tumor-tissue expression (Section 3.2) further complicate biological interpretation. Evidence from broader breast cancer populations, including findings for circulating RP11-445H22.4, cannot be directly extrapolated to early-stage TNBC [75]. For circRNAs, the available TNBC-specific literature combines preliminary clinical observations with mechanistic investigations [31,35,36], without establishing independently validated diagnostic performance in stage I–II disease. Similarly, the three-circRNA model of Yu et al. [65], developed in a general breast cancer cohort, was not evaluated separately for TNBC or stage I–II breast cancer. Overall, the reviewed evidence provides a stronger basis for further clinical evaluation of circulating miRNA signatures in early TNBC detection, while the diagnostic utility of lncRNAs and circRNAs requires more rigorous stage-specific validation.

Future studies should therefore move beyond exploratory case–control designs and evaluate circulating ncRNA biomarkers in prospective, adequately powered, multicenter cohorts that reflect the intended clinical setting of early TNBC detection. Similar priorities, including larger multicenter cohorts, harmonized reporting, transparent diagnostic cutoffs, and integration with imaging and clinicopathological variables, have been emphasized for circulating circRNAs in breast cancer [22]. Particular emphasis should be placed on the inclusion of well-characterized stage I–II TNBC cases together with clinically relevant comparator groups, including benign breast disease and other breast cancer subtypes, rather than relying predominantly on healthy controls [40,76]. Biomarker signatures and decision thresholds should ideally be predefined before validation, and their performance should be assessed in independent external cohorts using standardized protocols for blood collection, sample processing, RNA extraction, normalization, and analytical measurement [69,70]. For multivariable ncRNA panels, transparent reporting of model development and validation is essential to minimize overfitting and permit reproducibility across populations.

Importantly, future studies should also determine whether circulating ncRNA signatures provide clinically meaningful complementary value when integrated with established imaging and clinical assessment, rather than evaluating biomarker discrimination in isolation [40]. Ultimately, progression from retrospective diagnostic studies to prospective evaluation in populations representative of the intended screening or diagnostic setting will be required to determine whether the promising diagnostic signals observed for circulating ncRNAs can translate into clinically useful tools for early-stage TNBC detection [77].

Taken together, the available evidence supports circulating ncRNAs as promising candidates for the early detection of TNBC, but also highlights a substantial gap between diagnostic performance and clinical readiness. Among the evaluated ncRNA classes, miRNAs currently provide the most direct evidence in stage I–II disease, whereas lncRNAs and circRNAs remain less clinically validated for this specific application. Importantly, high diagnostic accuracy in small or selected case–control cohorts should not be equated with demonstrated effectiveness in real-world early-detection settings. The clinical potential of circulating ncRNAs will therefore depend not only on further improvements in diagnostic performance, but more importantly on reproducibility, methodological standardization, independent validation, and prospective evaluation in populations representative of the intended clinical use.

## 5. Conclusions

Circulating non-coding RNAs represent a promising source of minimally invasive biomarkers for the early detection of TNBC, but the available evidence remains insufficient for clinical implementation. Among the evaluated ncRNA classes, circulating miRNAs currently provide the most direct evidence, supported by studies demonstrating diagnostic discrimination specifically in stage I–II TNBC. In contrast, circulating lncRNAs and circRNAs have shown encouraging diagnostic potential, but their evidence is derived predominantly from general TNBC or breast cancer cohorts, subtype-discrimination studies, and relatively small exploratory populations rather than dedicated early-stage TNBC validation.

Importantly, high diagnostic accuracy alone should not be considered evidence of clinical readiness. The current literature is limited by heterogeneous study designs and pre-analytical and analytical procedures, frequent reliance on healthy control groups, small cohorts, and a lack of independent external validation. Future translation will require standardized assays and prospective, adequately powered, multicenter validation in well-defined stage I–II TNBC populations with clinically relevant comparators. Future studies should also establish whether circulating ncRNA signatures provide additional diagnostic value when integrated with imaging and clinical assessment rather than being evaluated as isolated biomarkers. Until such evidence is available, circulating ncRNA signatures should be regarded as promising investigational biomarkers rather than validated tools for early TNBC detection.

## Data Availability

No new data were generated in this study. All data discussed in this review are derived from previously published studies, which are cited in the manuscript.

## Abbreviations

The following abbreviations are used in this manuscript:

Abbreviation	Definition
AUC	Area under the receiver operating characteristic curve
CI	Confidence interval
circRNA	Circular RNA
EV	Extracellular vesicle
HC	Healthy control
HER2	Human epidermal growth factor receptor 2
lncRNA	Long non-coding RNA
miRNA	MicroRNA
NAC	Neoadjuvant chemotherapy
ncRNA	Non-coding RNA
RNA-seq	RNA sequencing
ROC	Receiver operating characteristic
RT-qPCR	Reverse transcription quantitative polymerase chain reaction
TNBC	Triple-negative breast cancer
TNM	Tumor–node–metastasis
